# Readiness of Sub-Saharan Africa Healthcare Systems for the New Pandemic, Diabetes: A Systematic Review

**DOI:** 10.1155/2018/9262395

**Published:** 2018-02-18

**Authors:** Bernardo Nuche-Berenguer, Linda E. Kupfer

**Affiliations:** ^1^National Institute of Diabetes and Digestive and Kidney Diseases, National Institutes of Health, Bethesda, MD 20892-1804, USA; ^2^Fogarty International Center, National Institutes of Health, Bethesda, MD 20814, USA

## Abstract

**Background:**

Effective health systems are needed to care for the coming surge of diabetics in sub-Saharan Africa (SSA).

**Objective:**

We conducted a systematic review of literature to determine the capacity of SSA health systems to manage diabetes.

**Methodology:**

We used three different databases (Embase, Scopus, and PubMed) to search for studies, published from 2004 to 2017, on diabetes care in SSA.

**Results:**

Fifty-five articles met the inclusion criteria, covering the different aspects related to diabetes care such as availability of drugs and diagnostic tools, the capacity of healthcare workers, and the integration of diabetes care into HIV and TB platforms.

**Conclusion:**

Although chronic care health systems in SSA have developed significantly in the last decade, the capacity for managing diabetes remains in its infancy. We identified pilot projects to enhance these capacities. The scale-up of these pilot interventions and the integration of diabetes care into existing robust chronic disease platforms may be a feasible approach to begin to tackle the upcoming pandemic in diabetes. Nonetheless, much more work needs to be done to address the health system-wide deficiencies in diabetes care. More research is also needed to determine how to integrate diabetes care into the healthcare system in SSA.

## 1. Introduction

While the international community is certainly aware of sub-Saharan Africa (SSA) problems caused by malnutrition and HIV and other infectious diseases, awareness of the increasing burden of chronic noncommunicable diseases (NCDs) such as diabetes is still low [1–3]. However, diabetes is not rare in SSA. It is estimated that, in 2014, 20 million people in SSA had diabetes and 523,000 died because of this disease or conditions related to it, with 76% of them under the age of 60 [4]. Moreover, the demographic changes coming with increased age and urbanization of the population favor a continued upward trend of diabetes in the region [5]. The prediction is that for the year 2035, 41.5 million people in SSA will have diabetes [4]. This increase is much higher than in any other region in the world [4]. In terms of prevalence, the limited data available shows that the prevalence varies from 3 to 15% [5, 6] with an overall diabetes prevalence for SSA in 2013 of 4.8% predicted to rise to 5.3% in 2035 [4]. In SSA, it is estimated that 62% of diabetic patients remain undiagnosed [4], which, if correct, represents the highest number of undiagnosed cases in the world. It is suspected that the very low prevalence of type I diabetes might also be an underestimate [7], due in part to the short life expectancy for children diagnosed with this disease, which varies between 7 months and 7 years depending on the country [6]. In general, the lack of proper surveillance systems for NCDs in LMICs makes the real magnitude of diabetes unknown and the lack of data makes it difficult to get policy makers' attention so that they can develop policies to prevent and address the increasing burden of diabetes on the population.

Due to the lack of attention to diabetes by both policymakers and healthcare professionals, patients usually present late in the diabetes cascade with complications of the disease [3]. Even when symptoms lead patients to seek care, they are often misdiagnosed [8]. From 2007 to 2010, it was estimated that due to untreated diabetes in SSA, millions of persons developed eye complications [9], blindness [6, 10], kidney damage [10], cardiovascular disease [10], or had lost a foot because of amputation due to diabetes [10]. It is therefore easy to infer that diabetes has a high social and economic impact in the region [11, 12] and affects employers and national economies due to the loss of productivity [13, 14].

Although the diabetes pandemic keeps growing, the estimated yearly expenditure on healthcare for diabetes in SSA in 2009 is the lowest of any other region in the world [4]. Moreover, the international organizations and NGOs that have successfully addressed other pandemics, such as HIV, have not yet begun to systematically address NCDs in the region. There is no doubt that to address diabetes, sub-Saharan Africa will require robust healthcare systems able to diagnose, treat, and provide chronic care for the diabetic patients. These systems should include the provision of diagnostic and treatment capacity, a trained workforce, and the existence of proper surveillance systems and treatment guidelines. In this review, we analyze the capacity of healthcare systems in the region to confront this new pandemic. We also identify examples and opportunities to integrate diabetes care into other successful, chronic care health platforms, specifically those devoted to care for people living with HIV (PLHIV) in a cost-effective way.

## 2. Methods

The data methodology used for this study was a systematic review that followed the Cochrane Systematic Review protocol [15]. The review took place between August and September 2014, and it was updated in August 2017. We searched for studies on the management of diabetes in countries of SSA. This included reports on diagnostic and treatment capacity for diabetes. We also sought for studies reporting interventions aimed to increase this capacity. Finally, we included in our search studies covering the topic of integration of diabetes care into existing HIV platforms.

### 2.1. Eligibility Criteria

We included articles published in English between 2004 and 2017 in peer-reviewed journals. We considered relevance to the review studies covering diabetes care in SSA countries, following the criteria of the International Diabetes Federation [4]. The rationale of the search was to obtain studies providing an overview of the current capacity to manage diabetes in SSA and the current attempts to increase capacity.

### 2.2. Information Sources

Databases searched for this review were selected based on their level of comprehensiveness and coverage of the topic; they included PubMed, Scopus, and Embase. Several gray literature sources were also considered. Expert opinion and snowball sampling techniques were then employed to identify other relevant documents that may have been omitted from the initial search.

### 2.3. Search Strategy

For the initial, broad selection, we combined the medical subject headings (MeSH) “diabetes,” “insulin,” “hyperglycemia,” and “type 1 or type 2 diabetes” and the region “sub-Saharan Africa” with terms such as, “health system,” “healthcare workforce,” “management,” “early diagnosis,” “treatment,” “non-communicable diseases,” “self-management,” “provider management,” “access to care,” and “RAPIA”(Rapid Assessment Protocol for Insulin Access). To cover the topic of integration of diabetes care into an existing platform, the HIV platform, we also combined all these terms with “HIV” and “PEPFAR” and “AMPATH,” two platforms supporting HIV care, prevention, and treatment in SSA.

### 2.4. Study Selection

A strategy was employed initially to identify English language studies that fulfilled a suitable combination of the following inclusion criteria: community-based; cross-sectional, randomized, cohort, case-control; reporting healthcare worker readiness for the management of diabetes; reported empowerment of patients for self-management of diabetes; and reporting efforts to integrate diabetes care in existing health infrastructures. In the first stage of the review, the initial selection of reports was screened and narrowed (see Table 1) to eliminate duplicates and those studies irrelevant to the main topic [16]. Then, the rest of the articles were screened based on the inclusion criteria. When multiple reports of the same study were retrieved, the most complete report was selected. Laboratory-based studies, anonymous reports, letters, commentaries, case studies, and reviews were excluded.

### 2.5. Data Collection Process and Data Items

After reading each article that appeared relevant and met the inclusion criteria, we made notes of the year of study and publication, objectives, study design, methodology, primary and secondary outcomes, location and year of study, population of interest, plan for future work, existence of national policies and/or guidelines for diabetes management, diagnostic and treatment capacity and availability for diabetics (availability of medicines, healthcare worker capacity), managing of diabetes care information (medical records), existence of linkage of diabetes care with care for other diseases, lessons learned from other disease platforms (e.g., PEPFAR) that could be applied to diabetes care, and recommendations issued by the authors. All the extracted data were reviewed by two independent researchers.

### 2.6. Risk of Bias in Individual Studies

To assess the risk of bias in each individual study, we collected the information on the limitations of each study as reported by the authors.

### 2.7. Summary Areas of Interest and Synthesis of Results

The principal areas of interest extracted from the studies were (1) diabetes diagnostic capacity; (2) access to medicines for diabetic patients; (3) capacity of healthcare workers; (4) pilot projects to enhance healthcare workers' capacity and (5) increase patients' adherence to treatment; (6) existence of electronic medical records; (7) provision of national guidelines for diabetes care; and (8) pilot projects to study integration of diabetes care into HIV and TB platforms. The results were synthesized and analyzed below following these areas.

## 3. Results

**Figure 1:**
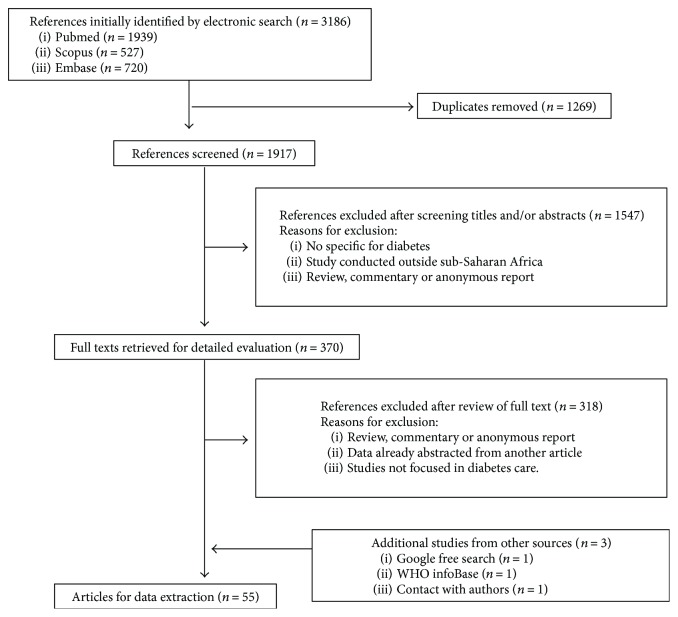
Flow diagram of the study selection procedure.

A total of 3186 English language records were initially identified, plus an additional 3 were included via snowball sampling and expert opinion. A title or title and abstract screening of the initial 3186 articles narrowed these down to 318 articles for full text review; finally, 55 articles remained that met the eligibility criteria by including evaluation of different aspects on health system performance, interventions devoted to the improvement of diabetes care, and some mention of the integration of diabetes care in other disease care platforms. These articles were included in the final qualitative analysis (Figure 1).

The degree of integration of diabetes within health systems varied from little to no mention of integration to examples in which significant integration of diabetes was explicitly discussed. Based on the study questions, nine areas related to diabetes and healthcare systems emerged from the analysis: (1) capacity to diagnose diabetes; (2) access to medicines for diabetic patients; (3) capacity of healthcare workers; (4) pilot experiences designed to optimize the effectiveness of healthcare workers' capacity; (5) pilot experiences for increasing patients' adherence to treatment (Table 1); (6) existence of electronic medical records; (7) provision of national guidelines for diabetes care; and (8) integration of diabetes care into HIV and TB platforms (Table 2).

### 3.1. The Capacity to Diagnose Diabetes

Diagnostic capacity is crucial for an effective healthcare system. Most of the literature analyzed in our review describes the poor diagnostic capacity of the healthcare centers studied in the region. Health centers studied in Mozambique [17, 18], Zambia [17], Rwanda [19], South Africa [20–24], Ethiopia [25, 26], Cameroon [27–30], Malawi [31, 32], Swaziland [25], Kenya [29, 33–36], Nigeria [29], Senegal [29], Uganda [37, 38], Ghana [39], and Tanzania [29, 40, 41] reported, especially in primary care facilities, a scarcity of glucose meters, testing strips, and laboratory capacity to measure glycosylated hemoglobin (HbA1C), which is the most reliable tool to diagnose diabetes. The lack of access to HBA1C testing can result in poor glucose control as reported in a cross-sectional study in Cameroon and Guinea [30]. Some initiatives, such as the “twinning program between the Mozambique and the UK government” [41], consisted in a series of activities supporting the Ministry of Health in developing a training program for specialists in diabetes care, developing patients education materials, establishing guidelines, and increasing the health system capacity. This program resulted in an increase in the availability of glucose measuring tools in Mozambique health centers [42]. In South Africa, the 1994 implementation by the Government of the diabetes management program improved diagnostic capabilities and currently some of the health centers, especially those in urban areas [22, 43], have diabetes diagnostic tools. Other countries like Cameroon [27, 37] have also improved their diabetes diagnostic capacity, but the general perception is that there is a lot of work to be done in SSA, especially in rural areas.

### 3.2. Access to Medicines for Diabetic Patients

Although there have been significant improvements, access to medicines in SSA is still limited. There are three main problems referred to in the literature: lack of availability in the health centers, large expense for the patients, and/or inadequate storage conditions. The best example of accessibility and affordability of diabetes medication is South Africa [20–23] where health systems went through a great transition after Apartheid, and the main diabetes medicines (it refers to those included in the WHO Essentials Medicines List: http://www.who.int/medicines/publications/essentialmedicines/20th_EML2017.pdf?ua=1) are now available and provided for free. However, it is common in SSA that government initiatives do not reach the rural areas [23, 44] resulting in poor access to medicines is these settings [23, 39, 44]. Cameroon has diabetes drugs on their list of essential medicines [28, 45], which should make them available and free to patients. In other countries like Zambia and Mozambique, insulin is sold free of taxes and available at a subsidized price [17]. However, procurement of insulin is poorly coordinated and often not adapted to the needs of the region resulting in improper storage during transportation and poor distribution to rural areas [17]. A study conducted in Uganda showed that only hospitals had an acceptable storage of diabetes drugs, while the local and subregional hospitals lacked first-line therapy for diabetes, and stock-outs were commonly reported [37]. In Mozambique, a partnership program with the UK government resulted in a more optimized system improving the procurement systems for insulin and therefore its availability [42]. In Tanzania, diabetes drugs are generally available but some reports show that about 36% of patients have problems affording the medicines [46]. Another study in Tanzania reports occasional unavailability of medicine in the public sector that forces the patients to buy their medication in the private sector at a higher price [40]. In Ghana, insulin is paid for by National Health Insurance; however, 45% of people are not registered and have to pay out of pocket for insulin [47]. Moreover, two surveys in Nigeria [48, 49] have shown that the economic burden of diabetes is high for some patients due to the impact of out of pocket payments. In countries like Rwanda [19, 50, 51], Ethiopia [25, 26], Swaziland [25], Kenya [52], Nigeria [53] Burundi, Senegal [54], and the Democratic Republic of Congo [51] diabetes drugs are still unavailable or are too expensive to be accessible. This is identified by patients as one of the biggest reasons for nonadherence to treatment [54].

### 3.3. Capacity of Healthcare Workers

The capacity of healthcare workers to manage diabetes is uneven in different countries. Medical doctors are often better capacitated than other healthcare workers such as nurses, nurse practitioners, medical officers, and nonmedical doctors [37, 40, 55, 56] to diagnose and treat diabetes. In countries like Mozambique and Zambia, low capacity of healthcare workers to diagnose diabetes have often led to misdiagnosis [17]. Another study in Cameroon demonstrated that healthcare workers had some problems in diagnosing diabetes, especially when using an HBA1C test, and did not have adequate knowledge about treatment, drug prescriptions, and management of the complications and comorbidities of the disease [57]. A survey in Uganda estimated that half of the healthcare workers had only fair knowledge of diabetes and that they generally had little experience in managing diabetic patients [37]. Another study in Uganda showed that healthcare workers had little knowledge of the use of HBA1C testing compared to fasting blood glucose (FBG) [58]. Similar results were obtained in a survey in Malawi [32]. In some of the selected studies, the healthcare workers with diabetes training do not fully understand the disease and may have misconceptions regarding treatment. For example, in a South African study, doctors did not know how to properly use/prescribe insulin and had fears related to the induction of hypoglycemia [20]. Another study in Nigeria showed how physicians were prescribing medicines that were not appropriate for their patients [56]. Although South Africa has a good health system overall, reports show that the primary care system is still poor and there is a need to empower the primary care centers in order to reach the rural areas [43]. Documentation exists that in Rwanda [19, 51], Nigeria [55], Mozambique [18], Ghana [39, 47], Uganda [38] Tanzania [40, 41], Burundi, and the Democratic Republic of Congo [51], there is little preparation or training of the healthcare workforce when it comes to dealing with diabetes and effective training programs are needed. This is probably true in many countries across the continent.

### 3.4. Pilot Experiences Designed to Optimize Healthcare Workers' Capacity

Several pilot interventions have been recently conducted to improve diabetes diagnosis and control capacity of healthcare workers in SSA. The training is given by doctors or by other qualified personnel. Most of these interventions are based on the idea of enhancing the training of the existing healthcare workforce and then transferring some of the functions (“task shifting” [59]) done by medical doctors to other healthcare workers. Task shifting has several benefits. First, it can alleviate some of the effects of the paucity of doctors available [60]; second, because community health workers (CHW) and nurses work in the community, it brings care closer to the patients, reducing the distance they must travel for treatment and so on; finally, it frees up doctors for more complex tasks that other healthcare workers cannot perform. In the context of diabetes, task shifting is focused on improving the healthcare workers' capacity for diagnosis, prescription of medicines, and education of patients about healthy lifestyles. The hope is that task shifting will result in higher rates of diagnosis and treatment adherence of diabetic patients. Pilot studies conducted in Ethiopia [26], South Africa [21, 23, 61], and Cameroon [27, 28, 62] have demonstrated this approach to be effective in increasing the use of testing equipment and patient retention. In Mozambique, after the implementation of a partnership program with the UK government, there was a significant improvement in the capacity of the healthcare workers for managing diabetes [42] and some studies have documented improvements in glucose control with patients living in areas where task shifting have been implemented [23]. A training program in Tanzania [63] successfully resulted in the elaboration of diabetes educational materials for patients, and a task shifting program with nurses in Kenya [64] showed nurses were capable of adhering to protocols and guidelines for diabetes care. An intervention in South Africa capacitating primary healthcare workers in identifying signs of diabetes and providing appropriate referral not only had an impact in this healthcare workers' knowledge but also improved early detection and referral for high risk, poorly controlled patients [43]. Approaches aimed at training pharmacists about diabetes management have also been successful [65]. In another pilot experience in South Africa [22], doctors received a capitation fee in advance for caring for diabetic patients and participant centers were responsible for any additional costs due to poor management. This achieved major reductions in hospital admission rates for acute metabolic emergencies and a sustainable reduction in patient's HbA1C [22]. Independent of the approach used, in general, the authors of these articles stress the importance of providing continuous education to the healthcare workforce. This is especially important to counteract the high turnover of personnel, especially in rural areas.

### 3.5. Pilot Experiences for Increasing Patients' Adherence to Treatment

Improving patient adherence to treatment is another key component for achieving optimal diabetes control in SSA. Lack of adherence could be due to several factors such as the high cost of medicines (see Section 3.1), poor care by healthcare workers (see Section 3.2), behavioral and environmental difficulties involved with following a healthy lifestyle, and/or the difficulty of getting to a health center [42, 66, 67]. Some studies suggest that access to care is more important than quality of care for the improvement of glycemic control [29]. Our literature review found some examples of pilot interventions that sought to increase adherence of patients to treatment by lowering some of the barriers to care—see Table 1. The aforementioned UK-Mozambique twinning program [42] was successful in improving adherence to treatment by increasing the information that patients got about diabetes and by increasing the access to diabetes drugs and diagnostic tools. The integration of diabetes care in primary care facilities, which reduces, in many cases, the problems of transportation, is also an effective strategy to improve patient retention rates. This has been demonstrated in several studies conducted in Cameroon [27, 66] and Kenya [33]. Another way to reduce the barriers associated with transportation was tried in Kenya and the Democratic Republic of Congo [52, 68] with the introduction of a cell phone-based home glucose-monitoring program although the clinical outcomes of these interventions have not yet been evaluated. However, in other studies, the introduction of home-based screening for diabetes did not improve the retention rates when compared to community-based screenings [34] and self-monitoring blood glucose (SBMG) did not result in better outcomes for patients in Nigeria [69], Kenya [35], and Cameroon [70]. Infrequent SMBG testing, lack of glucose meters, and limited patient involvement are all associated with the suboptimal glycemic control achieved by SBMG in these studies [70]. Other studies have proposed to lower the transportation barrier by integrating the linkage to care using mobile testing units [67].

A randomized control (RCT) study in Ghana [71] evaluating the effect of electronic reminders on risk management among diabetic patients proved this intervention successful in increasing adherence to medical appointments and reducing FBG in the intervention group. The establishment of peer-support groups has also proven effective in increasing diabetic patients' adherence to treatment and in improving clinical outcomes, as demonstrated by a RCT in Cameroon [72], by a retrospective study in Kenya [44], and in the setting of a diabetes camp for children in Cameroon [73]. Recently, an intervention in Kenya [36] extended the peer-support approach by including peer/microfinance groups and an integration of community education which resulted in significant improvements in linkage to care and clinical outcomes. In summary, linkage to and retention in care is very important for chronic diseases, and although it is especially difficult to achieve in rural settings, there are several interventions, such as peer support, mobile testing, and electronic reminders, that have been shown to be successful in addressing these areas.

### 3.6. Existence of Electronic Medical Records

Electronic medical records (EMR) are the foundation for complete population-based epidemiological information. EMRs, many of which track medicines used, also enables the prediction of the need for medicines avoiding the medicine shortages or “stock-outs” that are common in SSA. There are examples of the existence of EMRs in the region. One of the clearest examples is South Africa where all centers belonging to the Centers of Diseases and Endocrinology (CDE) network have access to a customized Internet clinical management program that they are obliged to use for entering the medical history of the patients [22]. In recent years, efforts to implement EMRs with diabetes modules have been conducted through pilot studies in several countries [25, 31, 51, 52]. EMRs have allowed the collection of epidemiological and financial data related to diabetes. Most of the studies identified by our review reported paper-based medical records. However, these records are easily lost and make sharing and aggregating information, as well as patient tracking, much more complicated.

### 3.7. National Guidelines for Diabetes Care

The standardization of protocols in the diagnosis and treatment of diabetes is another important aspect in achieving successful management of diabetic patients. The literature shows little prevalence of diabetes guidelines in SSA. Guidelines for diabetes care are found in South Africa [21, 22, 43, 74], Mozambique [42], and Cameroon [66]. However, sometimes, even when the guidelines are established, they are not disseminated to primary care centers like in Ghana and Tanzania [40, 47] or they simply do not take into account budgetary constraints making them difficult to implement [24]. In Uganda, the availability of guidelines for diabetes care seems to be unevenly distributed among the healthcare facilities at different levels as was demonstrated in a recent study where guidelines were found to be common in hospitals but not in lower level facilities [37]. Per another study in Uganda, it is also essential to establish specific cut-off points for HBA1C that consider ethnic differences [58].

The development and dissemination of treatment guidelines, appropriate to low resource settings, need to be addressed in SSA. Several of the pilots reviewed suggest that the development of guidelines is key to achieving successful diabetes care. As a basis for their guidelines, countries can use the WHO guidelines for prevention and control of NCDs: *Guidelines for primary health care in low resource settings (2012)*.

### 3.8. Integration of Diabetes Care into Existing HIV and TB Platforms

In SSA, there is a new opportunity to deliver chronic care for patients with diabetes through investments made in addressing other chronic diseases such as HIV and TB. A multicountry survey conducted in PEPFAR treatment centers showed how PEPFAR has laid the foundation for improving health system performance in health areas other than HIV, such as diabetes care [75]. In our literature search, we found reports of attempts to integrate diabetes care into the infrastructure built for care of other diseases and these findings are summarized in Table 2. For instance, in Malawi, efforts were conducted to use the infrastructure and also the lessons learned from a TB prevention and control program to also manage and monitor patients with diabetes [31]. In this program, diabetic patients, previously diagnosed, were also tested for HIV and TB, and the three diseases were managed in an integrated way. The program improved patients' knowledge about their condition, gave them access to treatment, and created a system of EMRs to track them over time. In Kenya, the innovative tools, structure, and interventions developed for the care of persons with HIV by the USAID-AMPATH program have facilitated the provision of basic diabetes care in the region: trained personnel, insulin, HbA1C, and point of care glucose testing devices have been added to HIV clinics [52]. This has revealed the poor quality of diabetes care, especially in insulin-dependent patients, and has helped to build a database of diabetics in the area. In Uganda [76, 77] and South Africa [67], community-based HIV testing campaigns also included screening and diagnosis for diabetes and other NCDs, which also, parenthetically, helped to reduce the stigma associated with persons who had HIV. This intervention identified new diabetic patients and the intervention in Uganda [76] achieved a 63% linkage of care to newly diagnosed diabetic patients.

The training of HIV health counselors to perform home-based diabetes and hypertension screening was also applied in Kenya [34] achieving success in the identification of new cases but a low yield (23%) in linking them to care. As a result of the success of this experience, the Kenyan Ministry of Health partnered with AMPATH and began the broad implementation of a diabetes- and hypertension-screening program [34]. Also in Kenya, hospitals ran by the NGO *Medecins Sans Frontieres* have been providing HIV-related services for several years, integrating hypertension and diabetes into their services to allow more holistic management of these diseases in a single patient [33].

However, the existence of a good HIV infrastructure by itself does not guarantee good provision of care for diabetic patients, as was demonstrated in two hospitals in Ethiopia and Swaziland [25]. In these two hospitals, despite a well-developed HIV infrastructure, only 26% of diabetic patients had optimal control of their disease. However, this same study demonstrated that after an intervention in the Ethiopian hospital, aimed to leverage HIV and diabetes services, important advances were made in increasing documented service delivery to diabetic patients and improving standards of care with no added staff [25]. Moreover, an EMR was generated based on the lessons learned from the existing HIV platform. This resulted in a general improvement in diabetic patients' attention and follow-up. However, as clinical outcomes were not measured for this intervention its comparison with similar initiatives is difficult.

## 4. Discussion

While the international community is certainly aware of SSA problems caused by malnutrition, HIV, and other infectious diseases, awareness of the increasing burden of noncommunicable diseases such as diabetes is still low [3–5]. It is “estimated” that, in 2014, 20 million people in sub-Saharan Africa had diabetes and 523,000 died because of this disease or conditions related to it with 76% of them under the age of 60 [4]. The prediction is that for the year 2035, 41.5 million people will have diabetes in SSA [4]. Thus, the number of diabetics in SSA will double in the next 20 years, an increase that is much higher than we see in any other region in the world [4]. It is important to highlight that the data on diabetes prevalence is based on estimations, since in 2009 only 15% of people with diabetes had been properly diagnosed [6] and although these numbers have improved significantly in 2013 [4], 62% of diabetic patients remain undiagnosed, which represents the highest number of undiagnosed cases in the world. Clearly, there is an urgent need to increase the capacity of health systems in SSA to manage diabetes.

**Figure 2:**
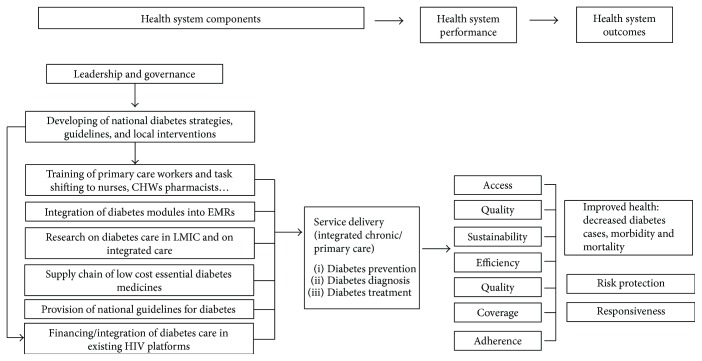
WHO health system building blocks based on the authors' recommendations.

Our review identified important flaws, but also successful interventions, in health systems in SSA that can affect care of diabetic patients. Diagnosis, access to diabetes medication, and trained healthcare workers are often unavailable, and EMRs and national guidelines for diabetes care are extremely limited. The combination of these factors explains the absence of necessary epidemiologic data, complicates tracking of patients, and hinders the ability to forecast future pharmaceutical and human resource needs [31]. Moreover, linkage and retention to care of diagnosed patients is hard because of the difficulties in the access to health centers, and the high prices or stock-outs of essential diabetes medicines [48, 49, 53, 66, 67]. Based on the findings in our review, we have modified the WHO health system building blocks (Figure 2) to represent the needs for effective management of diabetes in SSA.

In this review, we have found interventions that have been shown to be effective in SSA to address many of these issues. These include task shifting of healthcare personnel, reducing costs for and increasing access to care, ensuring the steady supply of drugs [17, 26, 40, 47, 55, 56], increasing diagnostic capacity [17, 34, 40], formulation, introduction, and adaptation of realistic clinical guidelines [24, 55, 58], implementation of diabetic modules for EMRs [31, 51], and education for both patients and healthcare personnel [17, 20, 33]. Especially in underserved areas [21], it is also recommended to train pharmacists or even traditional healers to tackle the lack of adequate healthcare workers to care for diabetics [18, 20, 23, 27, 28, 55, 65]. This training must be continuous to counteract the high turnover of personnel especially in rural areas [26, 56]. This can be achieved by increasing communication between doctors and other healthcare workers [43]. Some papers [78] recommend the creation of specific centers for the treatment of diabetes. However, it seems unrealistic to think that countries dealing with an unfinished agenda of infectious diseases should create vertical systems for the care of NCDs, especially because of the overlap in the continuum and chronicity of care of some of these diseases. Others recommend the establishment of partnerships with external organizations [19] or the integration of diabetes care in other disease platforms [25, 34, 40, 45, 52, 67, 76, 79]. One of the first efforts to achieve this integration was the application of the Directly Observed Therapy Short Course (DOTS) for tuberculosis which has been integrated to include diabetes care [79]. DOTS included political commitment, diagnosis, and standardized treatment by capable health workers, standardized monitoring and evaluation system, and uninterrupted drug supplies. The adaptation of this for diabetes in Malawi allowed the implementation of an electronic record system for diabetic patients [31]. This integrated model facilitates and improves care for patients while avoiding competition between communicable and noncommunicable diseases for limited resources. In fact, healthcare platforms should probably be designed by the chronicity of care given, chronic versus acute, rather than by the disease itself. An analysis conducted in centers funded through PEPFAR showed how the infrastructure originally built for HIV care can help to improve the health systems performance to care for other diseases [75] suggesting that this platform could be used for provision of more general chronic care. The integration of diabetes care in HIV platforms has been attempted [33, 34, 67, 76], has a very low cost (less than 3$ per patient) [76], and seems like a good option for sub-Saharan African countries.

A number of countries have developed national strategies to address diabetes. These strategies have included some of the interventions mentioned above, population-based screening [18, 23, 33, 34, 42, 52, 65], task shifting by training primary healthcare workers [21, 23, 26–28, 61, 62, 64, 65], provision of free medicines [20, 21, 28] or at a subsidized price [17, 46], and empowerment of patients [27, 33, 34, 66]. Partnerships have also helped. For instance, *Novo Nordisk* offers insulin at a lower price to LMIC countries [17] and several NGOs like *Medecines sans Frontieres* are including diabetes care in their facilities [33]. The Mozambique-UK partnership program has also achieved important advances in diabetes care for that country [42].

The studies included in this systematic review had some limitations, as mentioned by their authors. These limitations include low number of participants [28, 43, 63, 65–67, 73], participants and centers not representative sample of the studied country [25, 29, 40, 45, 53, 54]. Other studies reported high loss to follow-up [27, 50]. In general, the interpretation of the studies included in this review was difficult because of incomplete information, missing baseline data, and bias. Studies analyzing intervention data should be interpreted with caution due to the “regression to the mean” phenomenon [28, 66]. In interventions in poorly controlled patients, high baseline blood glucose values could explain the big reduction in blood glucose overestimating the effectiveness of the intervention [23]. Poor access to diagnostic devices and their lack of standardization might have underestimated the prevalence of diabetes [18, 29, 34]. In some of the cohort studies, the follow-up time was short [21, 44] and in general as some papers recognize [28] the pilot experiences need to be validated with randomized trials.

Further, while articles discussing both, health systems and diabetes in some respect were included in this review; it may have not always been the articles' explicit intent to consider this relationship. In addition, only articles written in English were included in the final pool of articles; this could have excluded some important information. As the relationship between diabetes and health systems in SSA has not been previously explored, the groupings that emerged during analysis were intended to guide discussion, but they themselves are somewhat arbitrary and further analysis may be useful. Finally, while the search criteria was as inclusive as possible and was supplemented by both a snowball sampling technique and expert input, some areas may have been unintentionally skipped.

About the methodology used to perform this systematic review, we followed the recommendations provided by the Cochrane handbook for systematic reviews [15]. This protocol is the most commonly used in the fields of medicine and health system interventions [16]. In this review, we did not perform statistical techniques (meta-analysis) to combine results of the eligible interventions. This was due to the lack of data about diabetes in SSA and also because the interventions included in our review, aimed either to optimize effectiveness of healthcare workers' capacity or to increase patients' adherence to treatment, were often using different approaches and were difficult to include in the same meta-analysis. That is probably the reason for the scarcity of meta-analysis measuring the impact of interventions aimed to improve NCD management in SSA [80]. For instance, a recent systematic review on the role of CHWs treating diabetes in the United States [81] suggests that CHW interventions have significant impact in physical health outcomes. However, similar analysis of the interventions that are currently being developed in SSA would be an important contribution to inform policy makers about effective interventions for the improvement of diabetes care in the region.

## 5. Conclusions

In summary, although improvements have been achieved in SSA in the last years for the provision of chronic diabetes care, many challenges remain. Improving the control and treatment of diabetes is a goal that SSA healthcare systems must achieve in the years to come, as the impact of the disease will grow steeply in the region. This objective must be accomplished in settings that still have an unfinished agenda for infectious diseases. That is why optimizing the existing resources by integrating diabetes care with other disease platforms, like those currently providing care for chronic diseases such as HIV and TB, is a great opportunity to improve diabetes diagnostic systems, provision of medicines, training of health personnel, empowerment of patients, and tracking of the disease burden. Finally, research into the most cost-effective way to implement chronic diabetes care in SSA is needed to successfully confront the upcoming pandemic.

## Figures and Tables

**Table 1:** Examples of interventions to improve patient adherence to diabetes treatment in SSA.

Country	Summary of intervention	Outcomes
Mozambique [42]	Improvement of care through establishment of partnerships and systematic care	Increased information about diabetes and access to care for patients
Rwanda [50]		
Cameroon [27, 66]	Integration of diabetes care into primary care facilities	Reduced transportation barriers and improved patient retention rates
Kenya [33]		
Kenya [52]	Cell phone-based home glucose monitoring programs	The clinical outcomes have not been evaluated yet
DRC [68]		
Kenya [34]	Establishment of home-based screening for diabetes	No improvement in clinical outcomes
Nigeria [69]	Introduction of self-monitoring blood glucose programs	No improvement in clinical outcomes
Kenya [35]		
Cameroon [70]		
South Africa [67]	Establishing of mobile testing units	Improvement in linkage to care
Ghana [71]	Setting off electronic reminders on risk management for diabetic patients	Increased adherence to treatment and reducing of FBG
Cameroon [72, 73]	Different approaches to establish peer support for diabetes patients	Increased adherence to treatment and improvement in clinical outcomes
Kenya [36, 44]		

**Table 2:** Examples of integration of diabetes care into other health platforms in SSA.

Country	Platform	Actions taken
Malawi [31]	TB-integrate HIV, TB, and diabetes	(i) Improved patients' knowledge (ii) Facilitated access to treatment (iii) Created EMR

Kenya [34, 52]	PEPFAR platform (AMPATH)	(i) Trained healthcare workers in diabetes care (ii) Improved diagnostic capacity (iii) Created a database of diabetics (iv) Created home-based diabetes screening programs

Uganda [76]	Community-based HIV programs	(i) Screening of diabetes and other NCDs (ii) Achieved a 63% linkage to care for diabetes

South Africa [67]	Community-based HIV programs	(i) Screening of diabetes and other NCDs (ii) Use of mobile testing units to reduce transportation barriers

Kenya [33]	MSF HIV platform	(i) Integrated management of diabetes and HIV (ii) Educated patients in healthy lifestyles

Ethiopia [25]	International Center for Diabetes Care and Treatment programs (ICAP)	(i) Applied lessons learned from HIV care to diabetes (ii) Established EMRs for diabetes
