# Erratum to “Readiness of Sub-Saharan Africa Healthcare Systems for the New Pandemic, Diabetes: A Systematic Review”

**DOI:** 10.1155/2018/3419290

**Published:** 2018-07-08

**Authors:** Bernardo Nuche-Berenguer, Linda E. Kupfer

**Affiliations:** ^1^National Institute of Diabetes and Digestive and Kidney Diseases, National Institutes of Health, Bethesda, MD 20892-1804, USA; ^2^Fogarty International Center, National Institutes of Health, Bethesda, MD 20814, USA

In the article titled “Readiness of Sub-Saharan Africa Healthcare Systems for the New Pandemic, Diabetes: A Systematic Review” [1], the format of Table 1 was unclear. The updated table is shown below.

## Figures and Tables

**Table 1:** Examples of interventions to improve patient adherence to diabetes treatment in SSA.

Country	Summary of intervention	Outcomes
Mozambique [42]	Improvement of care through establishment of partnerships and systematic care	Increased information about diabetes and access to care for patients
Rwanda [50]		

Cameroon [27, 66]	Integration of diabetes care into primary care facilities	Reduced transportation barriers and improved patient retention rates
Kenya [33]		

Kenya [52]	Cell phone-based home glucose monitoring programs	The clinical outcomes have not been evaluated yet
DRC [68]		

Kenya [34]	Establishment of home-based screening for diabetes	No improvement in clinical outcomes

Nigeria [69]	Introduction of self-monitoring blood glucose programs	No improvement in clinical outcomes
Kenya [35]		
Cameroon [70]		

South Africa [67]	Establishing of mobile testing units	Improvement in linkage to care

Ghana [71]	Setting off electronic reminders on risk management for diabetic patients	Increased adherence to treatment and reducing of FBG

Cameroon [72, 73]	Different approaches to establish peer support for diabetes patients	Increased adherence to treatment and improvement in clinical outcomes
Kenya [36, 44]		
